# Characterizing Human Perception of Speed Differences in Walking: Insights From a Drift Diffusion Model

**DOI:** 10.1523/ENEURO.0343-23.2025

**Published:** 2025-05-02

**Authors:** Marcela Gonzalez-Rubio, Gelsy Torres-Oviedo, Pablo A. Iturralde

**Affiliations:** ^1^Department of Bioengineering, Swanson School of Engineering, University of Pittsburgh, Pittsburgh, PA 15260; ^2^Center for the Neural Basis of Cognition, University of Pittsburgh, Pittsburgh, PA 15260; ^3^Departamento de Ingeniería, Facultad de Ingeniería y Tecnologías, Universidad Católica del Uruguay, Montevideo CP 11600, Uruguay

**Keywords:** decision-making, human locomotion, motor control, sensorimotor adaptation

## Abstract

Despite its central role in the proper functioning of the motor system, sensation has been less studied than motor outputs in sensorimotor adaptation paradigms. This is likely due to the difficulty of measuring sensation non-invasively: while motor outputs have easily observable consequences, sensation is inherently an internal variable of the motor system. In this study, we investigated how well participants can sense relevant sensory stimuli that induce locomotor adaptation. We addressed this question with a split-belt treadmill, which moves the legs at different speeds. We used a two-alternative forced-choice paradigm with multiple repetitions of various speed differences considering the probabilistic nature of perceptual responses. We found that the participants correctly identified a speed difference of 49.7 mm/s in 75% of the trials when walking at 1.05 m/s (i.e., 4.7% Weber Fraction). To gain insight into the perceptual process in walking, we applied a drift-diffusion model (DDM) relating the participants’ identification of speed difference (i.e., stimulus identification) and their response time during walking. The implemented DDM was able to predict participants’ stimulus identification for all speed differences by simply using the recorded reaction times (RTs) to fit a single set of model parameters. Taken together, our results indicate that individuals can accurately identify smaller speed differences than previously reported and that participants’ stimulus perception follows the evidence accumulation process outlined by drift diffusion models, conventionally used for short-latency, static sensory tasks, rather than long-latency, and motor tasks such as walking.

## Significance Statement

There is limited knowledge about the sensory information involved in the adaptation of motor outputs during locomotion. Therefore, we characterized the human ability to detect speed differences governing sensorimotor adaptation in split-belt walking, a well-known method inducing locomotor adaptation. Our unique approach revealed an enhanced sensitivity to smaller speed differences compared to previous studies. Additionally, we unveiled a mechanistic relationship between stimulus identification and RTs across speed differences of varying magnitudes in walking, using the DDM—traditionally associated with short latency, static sensory tasks—thereby highlighting the generality of the stimulus perception process across both static and dynamic tasks.

## Introduction

Humans possess a remarkable ability to adapt their movements when interacting with their environment. Through these interactions, the motor system compares expected and actual sensory consequences, allowing adjustments in subsequent movements (Shadmehr et al., 2010). In walking, the accurate integration of sensory information is essential for adapting to terrain changes and maintaining balance (Richardson and Hurvitz, 1995; Roll et al., 2002; Deliagina et al., 2008; Henry and Baudry, 2019; Qu et al., 2022). While motor outputs during locomotor adaptation have been extensively studied, sensation, particularly its role in walking adaptation, has received less attention.

A large body of research has explored the motor adaptations occurring during split-belt treadmill walking, where each leg moves at a different speed under various conditions (Dietz et al., 1994; Torres-Oviedo and Bastian, 2010; Mawase et al., 2014; Ogawa et al., 2014), and in diverse populations (Morton and Bastian, 2006; Finley et al., 2015; Sombric et al., 2017). However, the sensory contributions to these adaptations, specifically how people estimate leg speeds to generate appropriate motor patterns, are less understood. This gap likely arises from the inherent challenge of measuring sensation non-invasively. Unlike motor outputs, which have observable outcomes, sensation is an internal process.

Previous studies have typically assessed sensation during split-belt walking through active perception tasks, in which participants integrate sensory inputs and an efferent copy of the motor commands to self-report perceived speed differences between their legs (Jensen et al., 1998; Lauzière et al., 2014; Hoogkamer et al., 2015; Vazquez et al., 2015; Wutzke et al., 2015; Leech et al., 2018; Statton et al., 2018; Rossi et al., 2024b). Some of these studies have demonstrated that humans adapt their active perception of limb speeds in addition to their movements, highlighting the close connection between sensory perception and locomotor adaptation (Vazquez et al., 2015; Leech et al., 2018; Statton et al., 2018; Rossi et al., 2024a,b). However, these approaches often overlook the probabilistic nature of perceptual responses. Namely, individuals detect speed differences between their legs probabilistically, with accuracy depending on the magnitude of the difference. To clearly observe this probabilistic relationship, each sensory stimulus must be presented multiple times to each subject. Previous studies, however, have typically relied on only a small number of trials per stimulus (e.g., 1–3 trials), making it difficult to capture this effect. Additionally, many of these studies rely on yes/no tasks (e.g., “yes, there is a speed difference” or “no, there isn’t”), which can be heavily influenced by individual confidence levels. This can lead to substantial variability across subjects, as some individuals are more likely to respond “yes” when uncertain (Ehrenstein and Ehrenstein, 1999). To mitigate these issues, we propose using two-alternative forced-choice (2AFC) tasks, which reduce biases introduced by subjective confidence and provide a more reliable assessment of perceptual sensitivity (Goldstein, 2009).

Beyond detection thresholds, another valuable source of information, overlooked in walking studies, is reaction time (RT). RT, the time it takes for individuals to correctly identify sensory stimuli, provides insights into the sensory accumulation process during decision-making tasks such as 2AFC. To explore the relationship between RTs and perceptual accuracy, we employed the drift-diffusion model (DDM; Ratcliff, 1978; Bogacz et al., 2006; Gold and Shadlen, 2007). The DDM offers a principled approach to linking RTs and stimulus identification, treating them as expressions of the same sensory evidence-gathering process. This approach allows us to reveal underlying cognitive processes that may not be apparent through behavioral data alone (Myers et al., 2022).

In this study, we investigate how humans perceive speed differences between their legs during walking. We employ 2AFC tasks to characterize perceptual decision-making and describe the probability of stimulus identification as a function of belt speed differences. Additionally, we apply a DDM to analyze both stimulus identification and RTs, providing a unified framework to explore the sensory accumulation process during walking.

## Methods

### Data collection

#### Participants

A total of *N* = 39 healthy participants (24 ± 5 y.o., 22 female) completed the protocol. Four of the participants reported left-handedness and two reported being left-footed when kicking a ball (Kramer and Balsor, 1990). The protocol was approved by the Internal Review Board (IRB) at the University of Pittsburgh in accordance with the declaration of Helsinki. All participants gave written informed consent prior to testing.

#### Perceptual task

**Figure 1.**
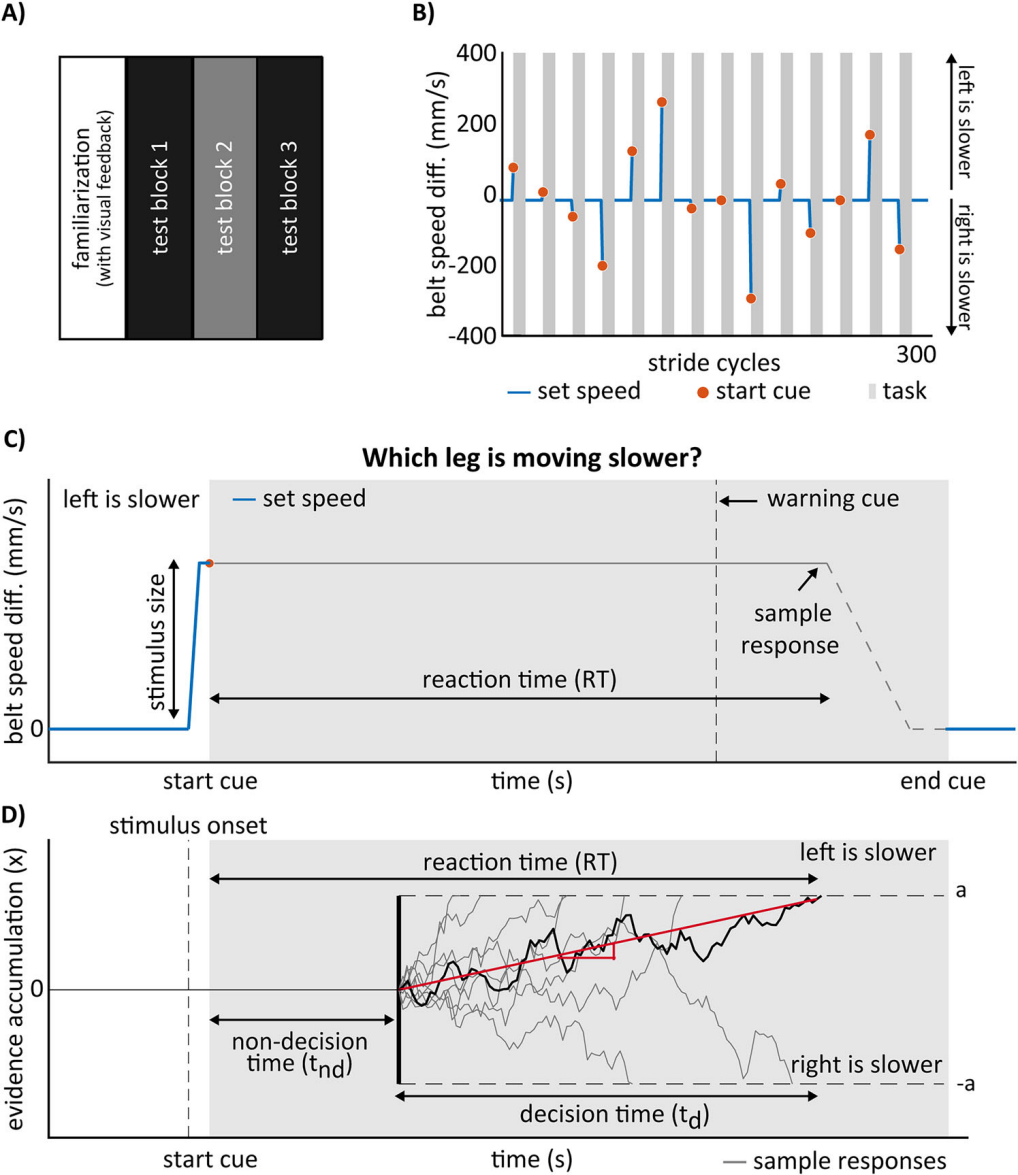
Protocol and methods for characterizing the perception of belt speed differences. ***A***, Experimental protocol. Participants completed a familiarization block followed by 3 testing blocks. ***B***, In each testing block, participants completed a series of perceptual tasks (indicated by the gray shaded areas). Specifically, Panel B shows one of the four sequences within a testing block. Each testing block includes four presentations of this sequence, either as a mirror image (where the belt speed differences are reversed) or with the presentation order flipped. As a result, each testing block comprises a total of four presentations for each non-zero stimulus magnitude and eight null trials. ***C***, Description of the perceptual task. The task began with an auditory cue (red circle in the task schematic). Upon hearing the cue, participants were instructed to identify the slower moving belt and make a keypress to indicate their response. In this study, we analyzed RTs and the choice made in the task. The task ended when the participant indicated a choice or after 8 strides of walking. ***D***, Schematic of the DDM. The DDM for 2AFC tasks represents the temporal evolution of a decision variable as a random walk (black and gray jagged lines). Decisions are made when one of the two decision barriers is reached (dashed lines: *a* and *b* = −*a*). RT is composed of a non-decision and decision interval. In this study, the non-decision time is defined as the interval between the auditory start cue and the onset of the evidence accumulation process. An example of the evolution of the decision variable is shown as the black jagged line, which has some drift rate (red line) and noisy evidence accumulation until reaching the upper threshold a, indicating that a decision has been made.

The perceptual task was designed to evaluate participants’ perception of speed differences, consisting on a 2-alternative forced-choice (2AFC) task (see Fig. 1, panel *C*). The task began with participants walking with both belts moving at 1.05 m/s, followed by an abrupt transition in belt-speeds to a speed difference (i.e., stimulus magnitude) whose value was unknown to the participants. The stimulus (Δ*V* = *V*_*R*_ − *V*_*L*_) was introduced by having one belt speed up by Δ*V*/2 while the other one slowed down by the same amount, such that the mean walking speed ( $\frac{V_{R}+V_{L}}{2}$) was maintained at 1.05 m/s throughout the experiment. The stimulus (speed difference) was introduced sequentially, with each belt changing speed when the corresponding foot is in the swing phase, starting always with the right belt. Subsequently, an audio cue signaled the start of the response window (depicted as the gray shaded are in Fig. 1*C*). This cue was provided at the beginning of the right leg’s swing phase following the update of both belts’ speed. The response window lasted a total of 8 strides (i.e., each stride duration was defined as the time interval between two foot landings of the same leg).

Upon the start cue, participants were required to press a key in either the right or the left hand-held controller, indicating which leg was perceived to be moving slower. No indication was given to prioritize either response speed or accuracy. The task concluded when the participant indicated a response or failed to answer within the 8 strides, signaled by a second, different, auditory cue indicating the task’s end. Participants did not perceive the belt’s acceleration during speed changes because the speed adjustments occurred when the corresponding foot was off the belt and not in contact with it.

Throughout the protocol, participants wore noise-cancelling headphones and a drape that obstructed their view of their feet. These measures were implemented to eliminate any auditory or visual cues that could potentially influence their perceptual responses. The headphones were also used to provide the start and stop audio cues, as well as producing an auditory message upon participants’ response that indicated their selection (e.g., “Left is slow”) and make participants’ aware that their response was recorded. Notably, the message did not provide any information on the accuracy of their response.

#### Testing protocol

The experimental protocol is illustrated in Figure 1*A*. Participants walked on a split-belt treadmill with an average speed across their legs of 1.05 m/s. To familiarize participants with the perceptual task, they completed 8 repetitions of the task while receiving visual feedback in the form of a live graphic displaying the speeds of each belt, as well as verbal feedback from the experimenters. During this familiarization phase, participants were encouraged to explore the task to understand the mapping between the controllers, the belt-speed differences, and all the auditory cues, regardless of response accuracy. This phase ensured they were comfortable with the procedure before data collection began. After Familiarization, participants completed 3 data collection blocks over two experimental sessions scheduled on two days within a week to avoid physical and mental fatigue. Each block consisted of 56 perceptual tasks, during which participants experienced a belt-speed difference (Δ*V* = *V*_*R*_ − *V*_*L*_) for a maximum of 8 strides (approx. 8 s, see Fig. 1*B*) interleaved with tied-belt walking (i.e., both belts move at the same speed). Within each block, all non-zero stimuli (±25, ±50, ±100, ±150, ±200, and ±300 mm/s) were presented 4 times each, while the null trials (0 mm/s) were presented twice as much (8 times). Stimulus presentation was in pseudo-random order and the same order was maintained across participants. The pseudo-random sequence was selected such that we ensured a balance between positive and negative perturbations minimizing potential biases introduced experimentally. The treadmill was stopped between blocks, and once within them, allowing participants to have breaks of about 3 minutes each. The first perceptual trial of each block after a break was omitted from the analysis due to participants frequently missing the initial 2AFC task immediately after the treadmill started, but the stimulus for this trial was collected at the end of each block to maintain the same number of repetitions per stimulus magnitude. In sum, a total of *M* = 117 blocks were collected and analyzed from the *N* = 39 participants (i.e., a total of 6,552 perceptual tasks, 56 perceptual tasks x 3 experimental blocks x 39 participants). Non-response trials within the blocks were removed (a total of 16 out of 6 552 perceptual trials or 0.24% of total perceptual trials), which were observed primarily for small speed differences (i.e., 5 trials of 0 mm/s, 5 trials of 25 mm/s, 3 trials of 50 mm/s, 2 trials of 150 mm/s, and 1 of 300 mm/s).

### Data analysis

#### Outcome variables from 2AFC perceptual task

We extracted three outcome variables from each presentation of the 2AFC task: (1) choice, (2) RT, and (3) accuracy. We defined a “left” choice as a response indicating that the left side was slower than the right side, and similarly, a “right” choice indicated the opposite. Reaction time was calculated as the time interval between the start cue and stimulus identification in the 2AFC task. Choice and RTs were calculated for all trials, including the null ones (i.e., 0 mm/s stimulus magnitude). Additionally, we converted the choices into accuracy scores, determining whether a response was correct or incorrect. An accuracy score had the value of 1 when the participant’s choice correctly indicated the belt that was moving slower, and zero when it did not. We did not calculate accuracy scores for null trials (i.e., 0 mm/s stimulus magnitude) since there is no correct choice in this case.

#### Point of subjective equality and just noticeable difference

We used the choice (left or right) responses in the 2AFC tasks to estimate two important quantities in the characterization of perception: the point of subjective equality (PSE) and the just noticeable difference (JND) also known as a difference threshold. The PSE refers to the stimulus magnitude at which participants exhibit an equal likelihood of choosing either option, resulting in a 50% choice rate for any of the two stimuli. This metric quantifies any potential biases in perception, such as a tendency to preferentially select the “left” more often than the “right” option, or vice versa. In healthy individuals, we would expect the PSE to be close to 0 mm/s, indicating that when both legs are moving at the same speed, participants are equally likely to choose either right or left. Further, we do not expect that the experiment will induce a bias, as the exposures to split-belt walking were short (8 strides or less) and inconsistent (i.e., both legs moved faster than the other at different instances of the perceptual task, without a predictable structure). The quantification of PSE is of interest because it has been used to quantify perceptual aftereffects following split-belt walking (Vazquez et al., 2015; Statton et al., 2018; Leech et al., 2018; Rossi et al., 2024a,b).

The just-noticeable difference (JND) represents the smallest difference between two stimuli that can be reliably detected by an observer. It is commonly used to quantify sensitivity to changes in physical stimuli in perceptual accuracy tasks (Salkind, 2006; Schwartz and Krantz, 2017). In this study, we define the JND as half the distance between the stimuli that produce 25% and 75% choice rates (Bush, 1963; Ulrich, 2010; Bausenhart et al., 2012; Aida et al., 2020; Paire et al., 2023). These choice rates are based on psychophysical practice, where responses at 25% and 75% represent key points on the psychometric curve. Specifically, the 25% choice rate marks where participants reliably choose one stimulus less often than chance, while the 75% choice rate marks where they reliably choose it more often than chance. This definition of JND is equivalent to averaging two separate sensitivity measurements. The first measurement captures the participant’s sensitivity below the point of subjective equality (PSE), calculated as the difference between stimuli that produce 25% and 50% choice rates. The second measurement reflects sensitivity above the PSE, calculated as the difference between stimuli that produce 50% and 75% choice rates. By averaging these two measurements, we obtain a more comprehensive assessment of the participant’s overall perceptual sensitivity across the stimulus range (Ulrich and Vorberg, 2009). Since the exact 25%, 50%, and 75% choice rates are unlikely to occur directly in the empirical data, interpolation is necessary to estimate both the JND and PSE. We applied a psychometric curve fitting approach described in detail below to infer the specific belt speed differences that correspond to the 25%, 50%, and 75% choice rates, from which the PSE and JND are then calculated based on the defined criteria.

#### Psychometric curve fitting

The psychometric curve fitting approach characterizes the relationship between participants’ choices and the associated stimulus magnitude. Specifically, we used parametric logistic regression to analyze participants’ choices. The responses were treated as samples drawn from a binomial distribution, where the probability of a particular choice (denoted as *p*) was modeled as a logistic function of a parameter *μ*_*j*_, as shown in Equation 1. In turn, the parameter *μ*_*j*_ depends on the stimulus Δ*V*, where *j* is each individual participant.

$$p=\frac{1}{1+e^{-\mu_{\;j}(\Delta V)}}.$$

In our analysis, we considered *μ*_*j*_ to have a simple linear relationship to the stimulus magnitude, as shown in Equation 2.

$$\mu_{\;j}(\Delta V)=\beta_{0j}+\beta_{1j}\Delta V.$$

We fitted the parameters for the two regressor logistic function (Eq. 2) in two ways: (1) to each individual *j*’s choices using a generalized linear fixed effect model, yielding participant-specific parameters *β*_0*j*_ and *β*_1*j*_. The method is equivalent to independently fitting a psychometric (e.g., logistic) function to each individual’s binary choice data using maximum likelihood estimation; and (2) using the responses from all participants with a generalized linear mixed model, with participant as the grouping factor for the random effects. In this approach, the model estimates the fixed effects ( $\bar{\beta_{0}}$ and $\bar{\beta_{1}}$), representing the mean *β*_0*j*_ and *β*_1*j*_ across participants, under the assumption that individual parameters are drawn from underlying Gaussian distributions. Parameters for the generalized linear mixed model were estimated using maximum likelihood with the Laplace method approximation. Notably, neither approach involved average data across participants. All model fitting was performed using the MATLAB’s (Mathworks, Inc., Natick, Massachussets, United States) *fitglme* function.

PSE and JND, defined above, were directly estimated from the two regressors *β*_0_ and *β*_1_ from Equation 2. Specifically, the intercept term (*β*_0_) reflects potential biases in participants’ choices. To estimate the PSE, we substitute Equation 2 into Equation 1, set the probability of choice to *p* = 0.5 (i.e., chance levels), and solve for Δ*V*. The relationship between our model parameters and the PSE for each participant *j* is:

$${\text{PSE}}_{j}=-\beta_{0j}/\beta_{1j}.$$

We can also solve for Δ*V* when *p* = 0.25 or *p* = 0.75 to quantify the JND. By doing so and plugging into the definition of JND, we obtain for each participant *j*:

$${\text{JND}}_{j}=\ln\;(3)\beta_{1j}^{-1}.$$

#### Statistical analysis

We used a significance level of 0.05 for all statistical tests. Additionally, to examine the relationship between JND, accuracy, and reaction times, we used Pearson’s correlation.

#### Secondary analysis

We also considered the potential impact of three exogenous factors on participant’s choices: habituation (Δ*V*_*prev*_), task learning (Δ*V* × *block*_[2,3]_), and laterality (|Δ*V*|). The full regression model including this factors is formally expressed:

$$\mu_{\;j}(\Delta V)=\beta_{0j}+\beta_{1j}\Delta V+\beta_{2j}\Delta V_{\;prev}+\beta_{3j}\Delta V\times block_{\left\{2,3\right\}}+\beta_{4j}|\Delta V_{\;j}|,$$

where Δ*V*_*prev*_ indicated the influence of the immediately preceding stimulus on the current choice. Δ*V* × *block*_{2,3}_ reflected whether the probability of choices changed over the course of the experiment, indicating a potential learning effect from exposure to the perceptual tasks. Lastly, |Δ*V*| identified a systematic difference in participants’ choices for stimuli of equal magnitude but opposite sign, potentially indicating asymmetric sensitivity between the legs.

### 2AFC decision-making as a drift-diffusion process

The DDM is a decision-making model that incorporates the accumulation of noisy evidence (e.g., Ratcliff, 1978; Gold and Shadlen, 2007). In the DDM, participants accumulate evidence over time until a sufficient amount of information is gathered, leading to a subsequent decision. Here, we considered the simplest form of DDMs for a 2AFC task (see Fig. 1*D*; Wagenmakers et al., 2007). The evidence gathered up to time *t* is represented by the continuous variable *x*(*t*). When *x*(*t*) goes above the decision barrier *a*(*t*), a left choice is made, and when it crosses below the decision barrier *b*(*t*), a right choice is made. In our DDM model, we assumed an unbiased starting point, which means that the starting point is equidistant from both decision barriers, indicating that participants have no preference for either response. Additionally, we assume constant decision barriers. Formally, the starting point of the decision variable is set at *x* = 0, and the barriers lie symmetrically such that *a*(*t*) = −*b*(*t*) = *a*.

The model separates the evolution of *x*(*t*) into two consecutive stages, as illustrated in Figure 1*D*. The first stage is the non-decision stage, which represents a delay (*t*_*nd*_) at the beginning of the evidence gathering process (Eq. 6). The non-decision time reflects early encoding of the stimulus as well as the execution of the motor response (Ratcliff, 1978; Shinn et al., 2020; Sandry and Ricker, 2022), which is external to the decision-making process (Myers et al., 2022).

$$x(t)=0,\;t\leq t_{nd}.$$

In the subsequent stage, the process of evidence accumulation is modeled as a continuous stochastic process with the following evolution (known as a Wiener or Brownian motion process with drift):

$$dx=r.dt+\sigma .dw,\;t>t_{nd}.$$

The variables *dx* represents the change in accumulated evidence during an infinitesimal time interval *dt*, while *dw* is a zero-mean normal process such that *dw* ∼ *N*(0, *dt*) for the same time interval. This equation describes a process in which evidence accumulates linearly over time with the given drift rate *r*, but is also influenced by additive noise accumulated over time with variance *σ*^2^Δ*t*. The evidence accumulation stage ends when the evidence accumulated reaches one of the decision barriers. The duration of evidence accumulation is referred as the decision time (*t*_*d*_), and the sum of the decision time and non-decision times gives the overall RT, which is the only directly observable time parameter (see Fig. 1*D*). We defined RT as the interval between the start audio cue (presented after the full speed difference was set) and the participant’s registered response.

This simple DDM is characterized then by four parameters: the barrier location *a*, which quantifies the necessary evidence to reach a decision; the diffusion rate (*σ*), which represents the rate of noise accumulation; the drift rate (*r*), which reflects the rate of evidence accumulation; and the non-decision time (*t*_*nd*_), accounting for processes like stimulus encoding and motor response. Although the model contains four parameters in total, it is scale-invariant, meaning that proportionally scaling *a*, *σ*, and *r* does not affect the model’s predictions. For simplicity, we set *a* = 1 without loss of generality (Wagenmakers et al., 2007).

Using the aforementioned simplification, the probability of the process reaching a specific decision barrier (e.g., the probability that a left choice is made, denoted *p*_*L*_, which corresponds to the positive or upper decision barrier; Fig. 1*D*) and the mean decision time have closed-form expressions (Bogacz et al., 2006; Wagenmakers et al., 2007). We adapted the expressions in Wagenmakers et al. (2007) to our parametrization of the problem and obtained the results shown in Equation 8 for the probability of making one choice, and Equation 9 for the expected RT. Equation 8 can be extended to obtain the probability of reaching the other barrier from *p*_*R*_ = 1 − *p*_*L*_.

$$p_{L}=\frac{1}{1+e^{\frac{2r}{\sigma^{2}}}},$$

$$E[t_{d}]=\frac{1}{r}(2p_{L}-1)=\frac{2}{\sigma^{2}}\frac{2p_{L}-1}{\log\;\left(\frac{1-p_{L}}{\;p_{L}}\right)}=\frac{2}{\sigma^{2}}\frac{\;p_{L}-p_{R}}{\log\;(p_{R})-\log\;(p_{L})}.$$

We note that Equations 1 and 8 are identical when substituting $\mu =-\frac{2r}{\sigma^{2}}$. Thus, this simple DDM can be thought of as the generative process for the logistic relation between stimuli and choices. Moreover, it provides a framework for connecting these quantities to RTs through the relevant parameters. We exploited this relation to extract predictions for choices, JND and PSE specifically, from RTs by fitting a DDM using only RTs.

Typically, the parameters of the Drift Diffusion Model (DDM) are fitted to the RTs of each presented stimulus individually. This approach is used when each stimulus is presented a sufficient number of times, allowing for a reliable estimation of the quantiles in the distribution of RTs (see e.g., Myers et al., 2022). We used a different approach because we sought to explicitly establish the dependence of the model parameters (*r*, *σ*, *t*_*nd*_) on the different sensory stimuli (i.e., speed differences, Δ*V* = *V*_*R*_ − *V*_*L*_). To this end, we adopted an approach similar to that of Palmer et al. (2005), specifically by making the following assumptions, which are common in the DDM fitting literature (Ratcliff and McKoon, 2008): (1) The non-decision time *t*_*nd*_ and noise (i.e., diffusion rate) are independent of the stimuli Δ*V*. (2) Evidence accumulation (i.e., the drift rate, *r*) in the task must vary with the magnitude of the stimulus (Δ*V*). The simplest such relationship is a linear one, which we choose to express as $r=\frac{\sigma^{2}}{2}(\beta_{0}+\beta_{1}\Delta V)$ for consistency with the nomenclature used in Equation 2. The model to be fitted then has four scalar degrees of freedom: *t*_*nd*_, *σ*, *β*_0_ and *β*_1_, and all previously presented relations in Equations 1 to 4 still hold for the DDM.

Parameters of the DDM were fitted to each individual separately by performing a least-squares fit to mean RTs using Equation 9. Estimates of PSE and JND were then calculated from the resulting *β*_0_ and *β*_1_. As there are two optimal fits with equal value but opposite signs for *β*_1_, the sign of *β*_1_ was imposed by bounding the optimization search interval for *β*_1_ to the positive half line. This is equivalent to enforcing that the optimal solution is one where participants are more likely to respond correctly for larger stimuli, rather than less. Resulting models are presented by averaging across all participants for visualization purposes.

### Code accessibility

The code/software described in the paper is freely available online at Open Science Framework (OSF), https://osf.io/9zh5b/.

## Results

### Characterization of somatosensory perception through the 2AFC task

Our objective was to characterize human perception of speed differences between their legs. To achieve this, we analyzed participants’ choices in the 2AFC task described in the Methods section. We (1) fitted individual fixed-effect logistic regression analyzes for each participant’s choices (Fig. 3*A*, light gray lines), using the presented stimulus (belt speed differences) as the predictor, and (2) performed a logistic regression with random effects grouped by participants to task choices across different stimuli magnitudes (Fig. 3*A*, black solid line).

### Individual differences in the point of subjective equality and just-noticeable difference

**Figure 2.**
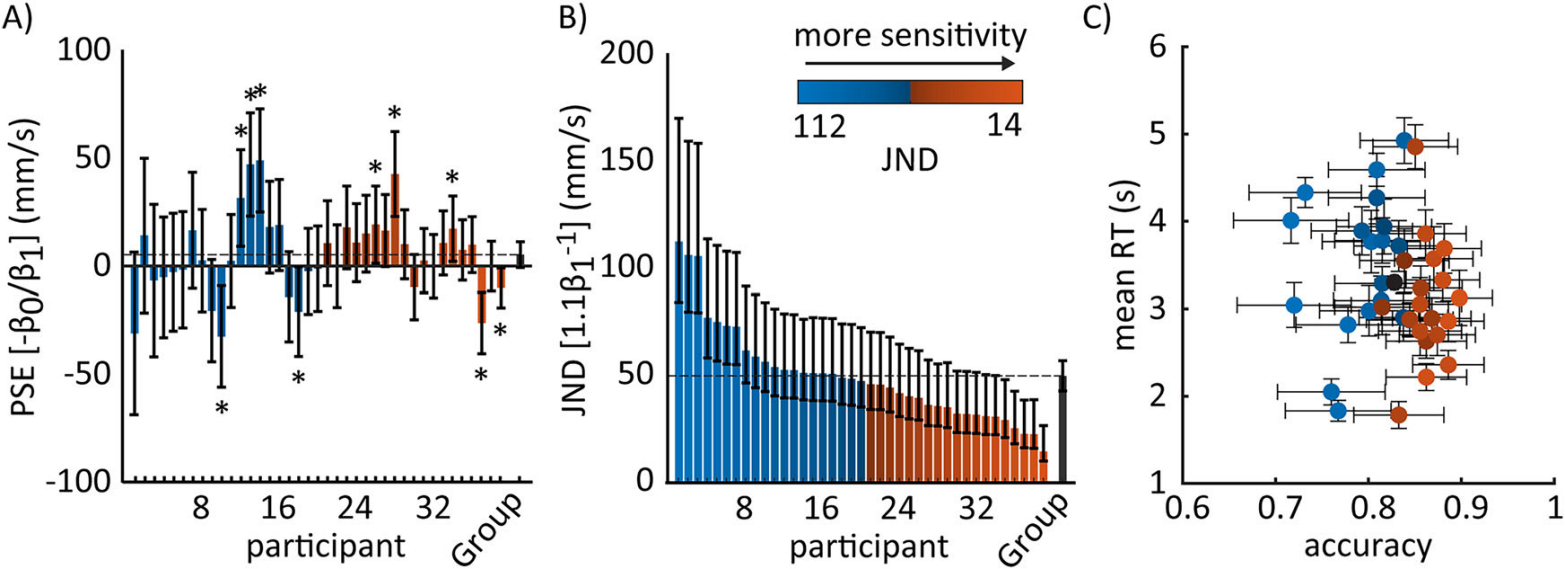
Individual variability in the perception of belt speed differences. The color in the circles and bar plots represent individual participants ordered by the magnitude of the JND. ***A***, Point of subjective equality (PSE) estimates are displayed as colored bars. Error bars illustrate the 95% confidence interval (CI) from the logistic regression models. The height of each bar plot and the error bars indicate the best estimate and approximate confidence intervals propagated from *β*_0_ and *β*_1_ (PSE = −*β*_0_/*β*_1_) presuming fixed *β*_1_ to its maximum likelihood value. Asterisks display significant PSE. The average of the individual PSEs is represented by the black bar height and the horizontal dashed line. ***B***, Just noticeable difference (JND) estimate with 95% CI (errorbars). Values are computed as 1.1/*β*_1_. Confidence intervals are computed by applying the same transformation to the edges of the CI of *β*_1_. This results in skewed CIs. Note that all the individual JNDs are significantly different from zero. The average of the individual JNDs are represented by the height of the black bar and the horizontal dashed line. ***C***, Mean RTs vs. mean overall accuracy across all stimulus magnitudes for each participant (± 1.96*standard error). The color of the dots corresponds to the JND values displayed in the gradient scale in panel B. The group average behavior is shown as the black data point.

**Figure 3.**
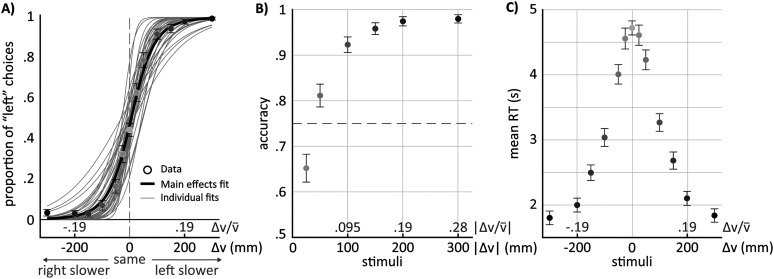
Choice, accuracy, and mean RTs as a function of stimulus magnitude. ***A***, Choice as a function of stimulus magnitude (Δ*V*, black labels) or stimulus magnitude scaled by mean walking speed ( $\Delta V/\bar{V}$, gray labels). Here, positive values indicate the left leg is moving slower, and vice versa. Circles indicate group average responses across participants, while the error bars indicate 1.96*standard error of the mean. The thin gray lines represent logistic fits to individual data (see Methods) and the thick black line represents the mean of the individual logistic fits. We used a generalized linear fixed model to fit the data of individual participants or a generalized linear mixed model with random effects. ***B***, Accuracy as a function of stimulus magnitude or stimulus magnitude scaled by mean walking speed. The data shown in the circles (group average ± 1.96*standard error) is equivalent to that shown in the left panel but averaged across positive and negative stimuli. Accuracy score was not calculated for the null trials. ***C***, Mean RT as a function of stimulus magnitude or stimulus magnitude scaled by mean walking speed. Circles indicate the experimental data (group average ± 1.96*standard error). Note that the color gradient in the circles among all panels depend on the absolute stimulus magnitude, which is a measure of task difficulty. Darker shades of gray mean easier tasks.

Individual participants exhibited a wide range of point of subjective equality (PSE) and just noticeable difference (JND) values. To determine individual PSE and JND, we performed individual fixed effect logistic regression analyses for each participant’s choices. Individual psychometric fits are shown as thin gray lines in Figure 3*A*. Figure 2 indicates the estimates for PSE (Fig. 2*A*) and JND (Fig. 2*B*) based on the regression coefficients from these individual psychometric fits.

Individual PSE values, which represents potential response biases, ranged from −32.5 to 49.1 mm/s, with an average of 5.3 ± 19 mm/s (mean ± standard deviation) across individuals (Fig. 2*A*). That is, on average, participants perceived the two belt speeds as equal when the right belt was 5.3 mm/s faster than the left one. We found that 10 out of 39 participants exhibited PSE that were significantly different from zero (Fig. 2*A*, marked with an asterisk) since the confidence intervals for the PSE values of these individuals did not bound zero. Thus, these participants had a significant bias, whereas the group did not.

The estimated sensitivity to stimulus magnitude, or JND values, ranged from 14.3 to 112.5 mm/s, with an average of 49.7 ± 22.6 mm/s (mean ± standard deviation) across the individuals. This means that participants required on average a 49.7 mm/s difference in belt speed to reliably identify a speed difference. We also express this JND as a proportion of the mean walking speed using the Weber Fraction (WF), defined as the ratio between the JND and the mean belt speed (i.e., $\Delta V/\bar{V}$ ). This yielded an average WF of 4.7%. The substantial range of values in bar heights for Figure 2*B* suggests that some individuals have much sharper discrimination curves compared to others.

Additionally, individual participants behavior varied considerably in their overall response accuracy and reaction times. Accuracy values ranged from 71% to nearly 90% (Fig. 2*C*, *x*-axis) and mean RTs across all stimuli ranged between 1.8 to 4.9 s (Fig. 2*C*, *y*-axis). We found no association between the measures after running a correlation analysis (*ρ* = −0.1347; *p* = 0.414). Conversely, there is a systematic relationship between JND values (Fig. 2*C*, colored scale) and overall accuracy of the choices (*ρ* = −0.942; *p* < 0.001). That is, participants with lower JND values were more accurate in their responses (orange cluster in Fig. 2*C*), indicating that higher perceptual sensitivity was associated with better performance on the task. Likewise, higher JND values correspond to poor accuracy in the task (blue cluster in Fig. 2*C*). In contrast, there is no clear association between mean RTs and perceptual sensitivity (*ρ* = 0.175; *p* = 0.286), as evidenced by the absence of blue or orange clustering (JND values) along the *y*-axis in Figure 2*C*. In other words, participants with similar JND values displayed varying reaction times.

### Group-level inference on PSE and JND using random-effects logistic regression

We performed a logistic regression with random effects grouped by participants, commonly referred to as a multilevel or hierarchical model, to analyze individual choices across the different tested stimulus magnitudes. In this approach, individual estimates are informed by data from the entire group, with population-level parameters offering a prior for individual-level estimations. We included this as a secondary analysis of group behavior because it provides less variable inferences for the individual estimates (Gelman and Hill, 2006).

We examined the potential influence of three external factors on participants’ choices with a full regression model (Eq. 5), in addition to each individual’s bias and sensitivity to speed differences (Δ*V*). Specifically, we considered habituation (Δ*V*_*prev*_), defined as the effect of the immediately preceding stimulus on the current choice; task learning (Δ*V* × *block*_2,3_), which assessed whether choice patterns changed systematically across experimental blocks due to repeated exposure; and laterality (|Δ*V*|), which tested for systematic differences in responses to stimuli of equal magnitude but opposite directions, potentially reflecting asymmetric sensitivity between legs. Our analysis revealed no significant habituation effect (Δ*V*_*prev*_, *p* = 0.194), indicating that participants’ choices were not influenced by the previous stimulus. Additionally, we found no evidence of task learning across experimental blocks (Δ*V* × *block*_2,3_, block 2: *p* = 0.06; block 3: *p* = 0.44), suggesting that participants did not systematically adjust their choices over time. Similarly, we observed no significant laterality effect (|Δ*V*|, *p* = 0.08), meaning participants did not exhibit asymmetric sensitivity depending on which leg was slower. As a result, only stimulus magnitude ( $\bar{\beta_{1}}=26\pm 1.5$, *p* < 0.001) and the intercept term ( $\bar{\beta_{0}}=-0.1\pm 0.07$, *p* = 0.056) were considered relevant factors influencing participants’ choices.

Based on these $\bar{\beta_{1}}$ and $\bar{\beta_{0}}$ values we found a PSE value of 5.5 mm/s with a 95% CI of [−0.1, 11.2] mm/s. This result is comparable to the reported average PSE from the fits from individual choices, which was reported to be 5.3 mm/s. In sum, the PSE lies between 5.3 and 5.5 mm/s. The group level sensitivity to stimulus magnitude, as indicated by the JND, was 44.4 mm/s (4.2% WF) with 95% CI of [39.8, 50.3] according to mixed effects model. These results suggest that the JND lies within the 4.2–4.7% WF range across the tested population.

When considering an analysis based on accuracy rather than choices, we observe that the 75% choice rate (Fig. 3*B*, gray dashed line) lies between 25 mm/s and 50 mm/s. This range aligns with the group level JND of 44.4 mm/s (reported above) and further supports the findings from the choice-based analyses. The correspondence between the approaches reinforces the robustness of the perceptual thresholds identified in this study.

Lastly, the averaged RTs for the group ranged between 1.8 and 4.7 s (Fig. 3*C*). The maximum mean RT was 4.7 s and occurred during null trials. Reaction times decreased as the size of the stimuli increased, regardless of the sign of the speed difference, eventually reaching a plateau at larger perturbation sizes. In other words, participants responded faster when the speed difference between the legs became more pronounced, regardless of which leg was moving slower.

### The DDM can explain both accuracy and RTs across stimuli in this task

A DDM was used to characterize the decision-making process in this task. Briefly, this model represents evidence accumulation as a random walk, where the drift is scaled according to the stimulus magnitude, and the noise (diffusion term) remains fixed across all stimuli (see Methods: 2AFC decision-making as a drift-diffusion process).

**Figure 4.**
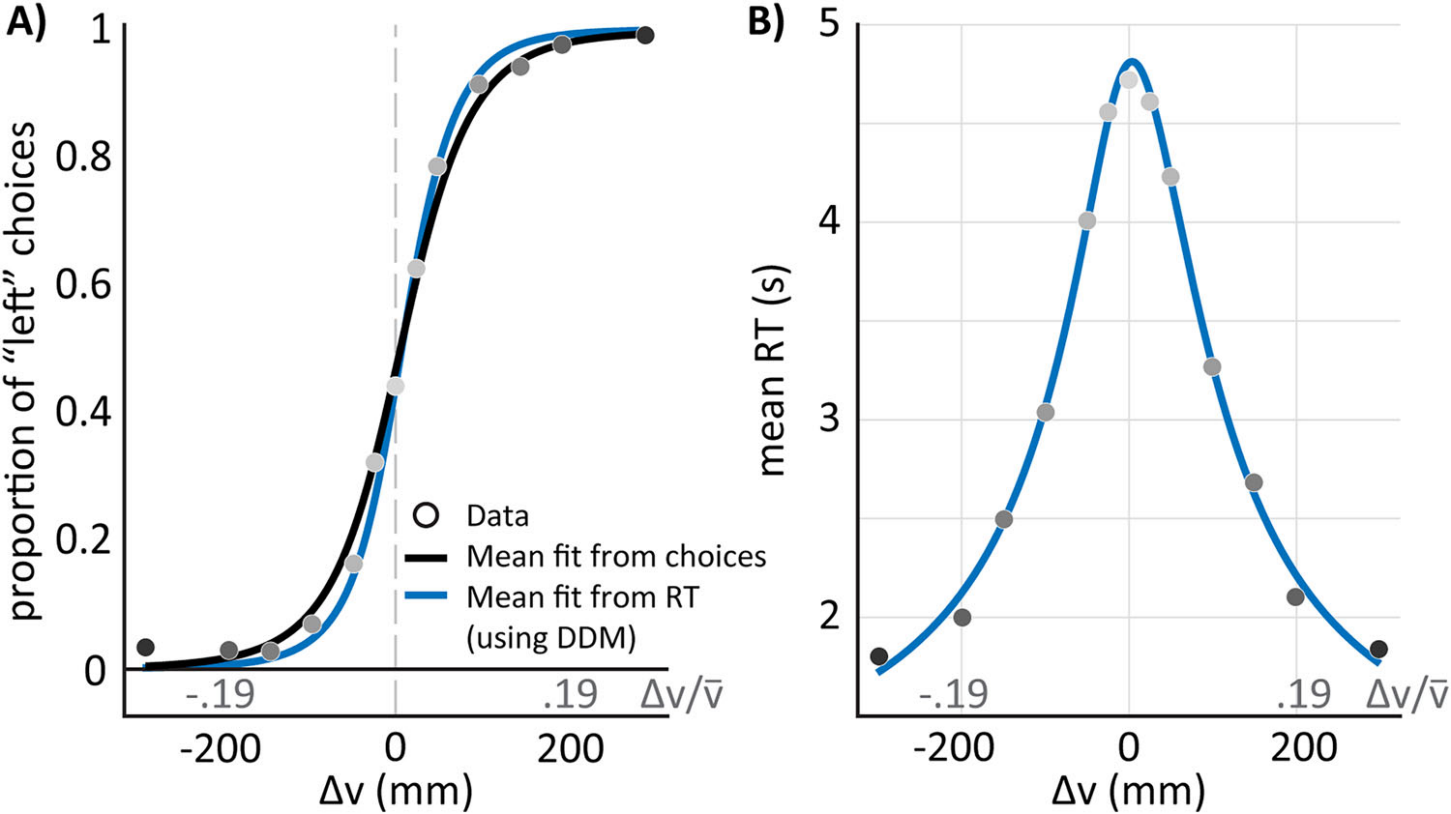
Drift-Diffusion model can predict choices based on RTs from the 2AFC task data recorded during walking. In all panels the circles represent the experimental data (group average). The color gradient in the data among all panels depend on the absolute stimulus magnitude, which is a measure of task difficulty. Darker shades of gray mean easier tasks. ***A***, Choices vs. stimulus magnitude (Δ*V*, black labels) or stimulus magnitude scaled by mean walking speed ( $\Delta V/\bar{V}$, gray labels). Black line represents the average of the individual fits to the choice data using generalized linear mixed models. This line is equivalent to that shown in Figure 3*A*. The blue line shows the prediction for the choices from the DDM fit on the reaction time data from panel B. ***B***, Mean RT vs. stimulus magnitude or stimulus magnitude scaled by mean walking speed. The DDM model was fit to each individual participant. The blue line represents the mean curve from fitting the RT data to each individual.

In this task, participants make their choice when the accumulated evidence reaches a predetermined value or threshold. Our findings showed that the experimental results could be effectively explained by this model (see Fig. 4). Thus, this mechanistic model relating RTs and choices captures the logistic relationship between stimuli and choice probability.

Consequently, we were able to predict participants’ psychometric curves (i.e., average choices) solely based on their RTs (Fig. 4, blue lines). From this analysis, we extracted PSE and JND values for each participant. The average PSE value across individuals was 7.1 ± 16.1 mm/s (mean ± standard deviation) and the averaged JND value was 36.5 ± 14 mm/s (or 3.5% WF). These values are qualitatively similar to the group’s values identified from the psychometric fits of the observed choices (Fig. 4, black line). Visually, the blue and black curves closely align, suggesting both capture the main trends in the data effectively. The group’s averaged values of the other model parameters were 0.5 ± 0.1 for the diffusion rate (*σ*) and 0.9 ± 0.7 s for the non-decision time (*t*_*nd*_). As we can appreciate from Figure 4*B* these four parameters characterize the RT changes as a function of stimuli magnitudes. Taken together, these results demonstrate that the DDM framework can extract meaningful information about perceptual sensitivity from RT patterns alone, providing a mechanistic perspective on the sensory evidence accumulation process during walking.

## Discussion

We conducted a comprehensive analysis using a novel and robust approach to characterize human sensitivity to differences in leg speed. Our larger dataset, compared to previous studies, and our distinct methodology suggest that humans are more sensitive to smaller speed differences than previously reported. Our results show that young participants are able, on average, to correctly identify the leg moving faster with 75% accuracy for differences of 49.7 mm/s or a 4.7% WF, which was our most conservative estimate for the group’s JND. In addition, we explored the relationship between participants’ choices and the time it took them to identify the stimulus (RTs). This relationship between choice and RTs was studied through a drift-diffusion model, which revealed that RTs in the 2AFC tasks that we used are informative about the identification of sensory stimuli during walking. Our study sheds new light on human sensitivity to leg speed differences and highlights the intricate interplay between stimulus identification and RTs across different stimulus magnitudes.

### Robust quantification of sensitivity to belt speed asymmetries in humans

In our study, we quantified sensitivity to asymmetric leg motion during walking by testing participants at various belt speed differences using a novel paradigm in this context. Specifically, we adopted a probabilistic approach to quantify perception through a symmetric 2AFC task, describing the likelihood that participants will correctly identify the belt with a higher or lower speed as a function of the size of the speed difference between the belts. Further, we show that the probability associated with the two choices in the task can be described accurately by a logistic regression over belt speed differences, which provides a comprehensive description of human sensitivity to belt speed differences while walking. To our knowledge, this is the first study to thoroughly report the whole probabilistic relation between participant responses and belt speed differences in the context of split-belt walking.

This probabilistic description highlights the variability inherent in perceptual experiences across a range of stimulus sizes and provides a natural framework for analyzing how different variables influence this perception. In our study, we use a logistic description of the perceptual responses that can be cast as a generalized linear model, allowing for the use of modern statistical tools to analyze the data. Future studies could investigate how aging, pathologies, or specific interventions lead to different choice rates or differences in any of the specific parameters that characterize the psychometric curve.

### Perceptual thresholds of belt speed asymmetries are smaller than previously reported

We are not the first to tackle the question of human sensitivity to belt speed differences while walking. Table 1 summarizes prior work on the topic and the corresponding main results. Our results stand out because we report a just noticeable difference (JND), or threshold, significantly below all previous reports.

**Table 1.** Summary of perceptual thresholds (JND) reported in the literature

Authors	Population	Procedure	JND
this study	Healthy young	symmetric 2AFC	4.7%
Hoogkamer et al. (2015)	Healthy young	y/n ascending limit	13%
Hoogkamer et al. (2015)	Cerebellar	y/n ascending limit	17.6%
Wutzke et al. (2015)	Chronic poststroke	y/n staircase	26%
Lauzière et al. (2014)	Healthy elderly	y/n ascending limit	12.8%
Lauzière et al. (2014)	Healthy elderly	y/n descending limit	16.2%
Liss et al. (2022)	Healthy young	y/n AFC slip task	6.9%

Perceptual thresholds are presented as a fraction of mean belt speed as a normalization procedure (Weber Fraction, $WF=\Delta v/\bar{v}$).

Given the different populations studied, some of the differences between our results and previous studies are to be expected. Such is the case of the studies by Lauzière et al. (2014) and Wutzke et al. (2015), and partially for the study by Hoogkamer et al. (2015). In all three cases, the populations studied are known to exhibit somatosensory deficits, such as older adults (Petrella et al., 1997; Thelen et al., 1998; Aydoǧ et al., 2006; Ribeiro and Oliveira, 2007; Yang et al., 2019; Shao et al., 2022), individuals with cerebral lesions (Carey, 1995; Connell et al., 2008; Tyson et al., 2008; Dukelow et al., 2010; Chia et al., 2019) or cerebellar lesions (e.g., stroke; Bhanpuri et al., 2012, 2013).

For healthy young participants, a single study has measured and reported the JND for belt speed differences (Hoogkamer et al., 2015). We found that the groups’s JND values for belt speed differences ranged between 4.2% and 4.7% WF, depending on the analysis we used to estimate them. These values are much smaller than the 13% WF previously reported by Hoogkamer et al. (2015). Even in the case of slip detection tasks, where the stimulus is a sudden change in belt speed and thus should be easier to detect, Liss et al. (2022) reported a JND of 6–7% WF for healthy young participants. We believe these disparities are rooted in the different methodologies employed, specifically in the use of a 2AFC task rather than a yes/no task.

All prior studies on the perceptual characterization of belt speed differences, regardless of the population, used variations of yes/no tasks. Our task presents participants with two symmetric alternatives (left and right choices). It forces them to choose between them, which is the preferred approach to objectively assess participants’ perception of physical stimuli and the underlying accumulation of sensory evidence (Green and Swets, 1966; Ulrich and Miller, 2004). The symmetric forced-choice approach has the advantage of not depending on the participant’s internal criterion for committing to a decision, and it has been shown that participants can make accurate reports about sensory stimuli even when they are not consciously aware of them, resulting in smaller detection thresholds (Ehrenstein and Ehrenstein, 1999). Further, in the context of yes/no tasks, the participant reports whether they perceived the stimulus or not, and the experimenter cannot validate the participant’s report externally. In contrast, in the 2AFC, participant responses to the presented physical stimulus can be evaluated as either correct or incorrect, providing an objective assessment of the participant’s ability to follow instructions, perform the task, and, ultimately, of the reliability of the measurement itself. We speculate that applying a 2AFC task in healthy elderly, cerebellar, or post-stroke populations would also lead to JNDs that are smaller and less variable across participants than those reported in the literature.

Conceptually, the JND is the smallest stimulus difference a participant can detect reliably. In our study, we operationalized the JND as half the difference between the stimulus levels at which participants’ choices were distributed at a 25% to 75% ratio between the two alternatives. This approach aligns with the *midpoint threshold* definition (Goldstein, 2009), where the JND is defined as the point midway between chance-level (50%) and the highest possible accuracy (100%), corresponding to 75% accuracy in our 2AFC task. Another widely used definition is the null hypothesis threshold, which defines the JND as the smallest stimulus difference that produces performance significantly different from chance levels (Goldstein, 2009). Although we did not adopt this definition in our study, we observed that choice rates differed significantly from chance-level performance for all stimuli tested. The smallest stimulus difference we tested was ΔV=25mm/s (2.4% WF), which resulted in 65% accuracy when pooled across all participants (*p* < 0.001). In a previous unpublished study, we also measured responses to a 10 mm/s stimulus (1% WF), which yielded above-chance performance as well (data not shown, Iturralde et al., 2020). Thus, if stimuli truly imperceptible to participants exist, they are likely smaller than 25 mm/s and possibly smaller than 10 mm/s. While these results provide robust evidence of participants’ perceptual abilities, future work could further refine the detection limits by exploring even smaller stimulus differences.

With regards to our PSE results, we found no systematic directional bias across the participant population. Only a minority of individuals (10 out of 39 participants) demonstrated significant biases that occurred in opposing directions, some participants consistently perceived their right leg as moving faster during minimal speed differences, while others showed the opposite tendency, favoring their left leg. Perceptual biases have been observed in lower limb, position-sensing tasks (Wood et al., 2023). This discrepancy may stem from the distinct proprioceptive mechanisms underlying speed perception versus position sense. Speed perception primarily relies on muscle spindle Ia afferents that respond to rates of length change, whereas position sense additionally engages group II afferents and joint receptors (Proske and Gandevia, 2012). The relationship between biases across these different proprioceptive domains warrants further investigation.

### Drift-diffusion models adequately characterize individual performance in the perceptual task

The DDM is a widely used framework for describing various perceptual tasks (Ratcliff, 1978; Gold and Shadlen, 2007), where participants make categorical decisions based on the accumulation of noisy sensory evidence. Specifically, it describes the decision-making process of discriminating between two different sensory stimuli. In these tasks, participants must integrate sensory evidence over time to favor one of the alternatives when presented with a choice (Bogacz et al., 2006). A decision is reached when sufficient sensory evidence is accumulated favoring one choice. The accumulation of evidence is believed to reflect the integration of sensory information during a 2AFC task (Ratcliff, 1978; Gold and Shadlen, 2007; Ratcliff et al., 2016; O’Connell et al., 2018), establishing a clear relationship between choice and RTs. Our study used the DDM to examine the relationship between stimulus identification and RTs across speed differences of varying magnitudes in walking. This approach aims to gain insights into the underlying sensory integration process in the decision-making task.

We discovered that a DDM formulation in which the evidence accumulation rate varies linearly with the stimulus magnitude, while all the other parameters remain fixed, can characterize both RT patterns and predict choice behavior across all stimulus magnitudes. This demonstrates the utility of the DDM as a mechanistic framework that connects RTs to the underlying perceptual decision-making process, rather than as a statistical alternative to psychometric function fitting. By applying the DDM to our walking task, we reveal that the sensory evidence accumulation process that has been well-characterized in static perceptual tasks is also operational during dynamic tasks like walking, supporting a common mechanism for perceptual decision-making across different sensory contexts.

Notably, DDM parameters across different stimulus magnitudes are typically fitted separately, without assuming any specific scaling between stimulus magnitude and DDM parameters, but require working with the entire distribution of RTs (Ratcliff and McKoon, 2008). However, here, we adopted a unified framework where the DDM parameters are described as a function of stimulus magnitude, similar to that of Palmer et al. (2005). This approach yields the surprising result that a single set of DDM parameters per participant, extracted solely from average RTs across stimuli, can reproduce the choice data well. This suggests that RTs for the different stimuli are informative about the availability of sensory information needed to identify a speed difference between the legs accurately.

The DDMs were developed for tasks with fast RTs, usually involving passive sensory perception, where decisions are made within less than 1.5 s (Swensson, 1972; Tanaka and Shimojo, 1996; Roitman and Shadlen, 2002; Huk and Shadlen, 2005; Balslev et al., 2007; Gandhi et al., 2013; Reinagel, 2013; Pardo-Vazquez et al., 2019; Dekel and Sagi, 2020). More recently, Lerche and Voss (2019) extended their use to moderately complex tasks with RTs of up to 3 s. In our study, we further validate the use of DDMs for tasks with even longer decision-making durations, where RTs ranged from 1 to 10 s. The implications of using DDMs to model decision-making with longer RTs are significant. In tasks with short reaction times, the DDM model parameters have been linked to neural decision-making substrates (Gold and Shadlen, 2007; Ratcliff and McKoon, 2008; Ratcliff et al., 2016; O’Connell et al., 2018), and our findings open the door to exploring these neural and behavioral mechanisms for extended decision contexts, like the one presented in this study. For example, previous studies have shown that stressing accuracy or speed alters specific DDM parameters and that some empirical observations, such as unequal distribution of RTs in correct vs. incorrect trials, may arise because of variability in the parameters that characterize the evidence accumulation process (Ratcliff and McKoon, 2008). Future studies should investigate the presence of these effects in the context of our task and whether the DDM can be modified to account for them.

In our DDM implementation, we defined RT as the interval between the auditory start cue and the participant’s response, rather than from the initial belt speed change. This decision was based on our experimental design, where the complete stimulus (the full speed difference between legs) is only available after both legs have experienced their respective speed changes. While the stimulus onset technically begins with the first leg contact following a belt speed change, participants cannot make informed decisions about the speed difference until experiencing both speeds. The interval between the first belt speed change and the start cue was consistently short (on average 0.73 s) and remarkably stable across stimulus magnitudes (0.03 s standard deviation across stimulus magnitudes, averaged across subjects). This means our choice of time reference would only shift the non-decision time parameter by a subject-specific constant value without affecting other model parameters. Our operational definition anchors measurements to a clearly defined event (the auditory cue) while maintaining the validity of our DDM analysis.

DDMs offer valuable insights into the workings of the nervous system despite the lack of neuronal recordings in behavioral studies, such as the one presented in this study. For instance, Pardo-Vazquez and colleagues used this model to make inferences about the neural coding of sensory stimuli across varying stimulus magnitudes (Pardo-Vazquez et al., 2019), which aligns with our research protocol. They combined a decision-making model and 2AFC tasks to assess auditory perception of different stimuli combinations. They found that the stochastic nature of the neural encoding of stimuli provided a mechanistic explanation for Weber’s Law–a common empirical observation across sensory systems (Zanker, 1995; Durgin et al., 2009; Contu et al., 2016; Pardo-Vazquez et al., 2019). Notably, this outcome does not hinge on specific assumptions regarding stimuli or task characteristics, implying potential generality across sensory modalities and tasks. Future studies are required to investigate the presence of Weber’s Law regularity in dynamic tasks such as walking.

Our findings suggest that humans have greater sensitivity to leg speed differences than previously known. Moreover, we have demonstrated that a DDM can explain the relationship between stimulus magnitude, RTs, and choice in a decision-making task. These results hold significance within the framework of sensory perception and decision-making during a dynamic task such as walking, providing valuable insights into human sensitivity to leg speed differences and the underlying processes at play. The study’s methodology and findings contribute to our understanding of how humans perceive and discriminate speed differences during locomotor tasks and can potentially be applied to studies investigating the effects of aging or brain lesions on sensory perception.

## Synthesis

Reviewing Editor: David Schoppik, New York University Grossman School of Medicine Decisions are customarily a result of the Reviewing Editor and the peer reviewers coming together and discussing their recommendations until a consensus is reached. When revisions are invited, a fact-based synthesis statement explaining their decision and outlining what is needed to prepare a revision will be listed below. The following reviewer(s) agreed to reveal their identity: Jonathan Wood.

Major comments

1. The primary hypothesis: "there is a minimum speed difference that people can consistently perceive" is not falsifiable, as there is always a minimum detectable threshold across sensory systems. We recommend reframing the work as an exploratory study rather than a hypothesis-driven study.

2. Clarify the term "sensation". The measurement of velocity difference during walking is not a pure assesment of sensation. There are likely motor components involved because individuals are actively walking. For example, an efference copy helps in the decision-making process since active movements are being performed. At the very least please specify exactly what is meant by the measurement of velocity perception (i.e., what do the authors assume goes into this measurement?).

3. AFC task methods.

a. We are concerned that allowing individuals to adjust the speeds of the belt after the AFC decision was made could have provided them feedback about their performance. This is problematic as it could have influenced future decisions, inducing learning. We recognize the term to control for a practice effect in the logistic regression model. While these practice effect terms did not impact the probability of responding left, they still could have influenced accuracy. To ensure learning did not occur, is it possible to determine if accuracy of participants responses changed across trials or blocks? If there was no learning, accuracy should be similar across the blocks. Additionally, it could help to look at improvements across trials during the familiarization block, when they were provided with actual feedback, and compare familiarization block accuracy improvements to accuracy improvements during the task itself.

b. We believe each trial was only presented 2-4 times for an individual. In the introduction (lines 56 - 59) the manuscript highlights the importance of considering the probabilistic nature of responses when the same stimuli are presented multiple times. To support the line in the introduction, please explicitly evaluate whether 2-4 trials are not enough to accurately estimate the PSE and JND, and if not, detail this limitation in the Discussion.

c. It is good that you interleaved tied belt walking trials between the split belt trials (line 146). But how long were the tried belt trials? Based on the figure it looks like they were also about 25 strides? We assume this was to prevent any motor adaptation from developing during the split belt trials, but motor adaptation could have an effect much longer than 25 strides. Is it possible to separate the potential motor effects of the perturbation from the sensory perception? Please address this concern in the Discussion.

4. AFC task analysis and statistics.

a. We am confused why you started with a logistic regression model to obtain PSE and JND values. The simplest way to estimate PSE and JND values would be to fit a psychometric function to each individual's data. Then the PSE and JND values can be calculated individually from these fits. Since the only two terms that were meaningful was the stimulus magnitude and intercept and it may be that the JND estimates derived from this method may have underestimated the JND (line 385). We suggest fitting each individual's data using psychometric function (a logistic or cumulative normal). This way, the PSE is simply the point where the probability of responding left = 0.5 and the JND is the difference between 0.75 and 0.25.

b. We can see the value of performing the logistic regression as a secondary analysis to confirm that the only meaningful predictors for participant responses was the stimulus position, but to do so, the manuscript should also consider each individual's bias by adding a term for subject into the model when you fit this model to the pooled data.

c. Definition of JND. Why does the manuscript adopt the midpoint definition of JND? This definition is generally reserved for studies that assess an absolute threshold of detecting a stimulus e.g. a set of stimuli with speeds from 0 to x stimulus magnitude. The way the task in the manuscript is set up, specifically with the stimulus positions being both positive and negative, seems to lead to an analysis where the JND is the difference between the 75th and 25th percentile.

5. Drift diffusion model.

a. The manuscript would benefit from explication on what specific and unique insights the drift diffusion model provides into the sensory process. In the introduction (paragraph starting on line 70 and lines 92-94), it seems the drift diffusion model is being used to determine the relationship between response time and accuracy (the speed accuracy trade-off) in this task. But that goal could be accomplished with a behavioral analysis rather than the model. In the introduction, it would be helpful to provide an intuitive rationale for the use of the DDM and the specific and unique insights this model could provide, if any.

b. Why does sigma increase along with increasing magnitude of the stimulus? We would expect decision uncertainty (or diffusion) to decrease as the stimulus level increased. It sounds like this has been observed before (based on the discussion starting in line 600), but no explanation for why this happens here is provided. It may help to answer the following questions: since the DDM model is supposed to represent the neural underpinnings of the decision-making processes, why is this specific task prone to magnitude-dependent noise? And what does that tell us about velocity perception while walking?

c. Generally, the impact of this work would be helped tremendously by providing a clear interpretation for the findings in this model and how they relate back to the detection and perception of speed differences while walking.

d. What is the principal behind the non-decision time parameter? Is it necessary for the model? Wouldn't individuals immediately start to gather information to make their decision as soon as the signal notified them to? Consequentially, we are not sure what the non-decision time is supposed to represent in terms of sensory processing.

Minor comments

6. Introduction: It would be helpful for the reader to understand why it is important to have accurate and precise estimates of limb speed (balance, split-belt adaptation, etc.). Opening the introduction with adaptation gives the impression velocity sense differences will be assessed pre and post adaptation.

7. Introduction (Line 47): Consider adding Morton et al., 2006 to references for the cerebellar population.

8. Yes/no tasks are comparable to 2AFC tasks contra lines 64-69 because a yes/no task is still a type of 2AFC task.

9. Introduction (line 94-95): Before its ability to be used as normative data, the reliability of this method should be examined. Also, many participants would be necessary before we assume these data are normative.

10. Methods/results: are the results of the 2AFC task consistent with the results of the speed matching task? That is, is the bias similar between the two tests? If so, this would validate the current method.

11. What was the acceleration on the belt when the split occurred? It could be either a very slow change or a faster and more abrupt change if the acceleration of the belt was set high. A faster acceleration might make it easier to detect a difference. What effect do you think acceleration had on participant responses?

12. Figure 1D. Please describe each component of the figure in the figure legend. The red line is not described. What is the difference between the black thick and smaller thin lines?

13. Results (line 352): Here the manuscript clearly states the purpose of the study: "In this study, we wanted to determine which factors influence human perception of speed differences between their legs." But this was not mentioned as the purpose in the intro.

14. Results (line 365): In this sentence, it comes across like the intercept result is on equal footing as the stimulus magnitude result. However, the beta values show that the stimulus magnitude clearly has a larger effect.

15. Results: please provide an intuitive interpretation for the JND and PSE results for the reader. For example, what does a 94 mm/s JND mean in the context of the task? For example, consider "it takes a 94 mm/s increase for the individual to detect a difference in speed." Similarly for the PSE result.

16. Results (line 407). Please cite the previously reported group level JND estimates.

17. Discussion (lines 469 - 471). Please expand on the potential differences in methodology that could have led to the differences in JND between the current study and others.

18. Discussion (line 508). How do we know that left, right responses in the task have symmetric value? Could the (small) bias observed in your study, or other studies that employ the left/right response (Vazquez et al 2011) be explained by a preference for one response over the other? In your study all the individuals were right limb dominant, could that have influenced responses? We recommend caution in using this specific explanation as a justification for the benefits of the current method.

19. Discussion (lines 571 - 573). Please explain some potential reasons why the traditional formulation of DDM is inadequate for describing the decision-making process in this task.

20. In general, the discussion is focused on the methods. It would be more impactful if the discussion was focused more on the findings themselves. This is especially true for the DDM discussion which would be helped by linking components of the DDM and the variations employed in the current study to the neural underpinnings of the decision-making process in the context of the current task compared to other decision making tasks.
